# Increasing fetal hemoglobin as a possible key for improvement of hypoxia and saving last breath in COVID-19 patient: “postulating a hypothesis”

**DOI:** 10.1186/s43168-021-00078-7

**Published:** 2021-06-24

**Authors:** Muhamed A. Abdelzaher, Ashraf E. S. Ibrahim, Essamedin M. Negm

**Affiliations:** 1 Neurosurgical Department, Alahrar Teaching Hospital, Zagazig, Egypt; 2 Chest Department, Faculty of Medicine, Zagazig University, Zagazig, Egypt; 3 Anesthesia and Surgical Intensive Care Department, Faculty of Medicine, Zagazig University, Zagazig, Egypt

**Keywords:** COVID-19, Hypoxia, Fetal hemoglobin, Fetal blood, Hydroxyurea

## Abstract

**Background:**

COVID-19 patients normally experience mild cold-like symptoms that progress from the early viral response phase through the lung phase to the hyper-inflammation phase. Acute respiratory distress syndrome (ARDS) characterizes the most critical stage of the illness with progressive respiratory failure. Hypoxemia is the most dangerous and challenging problem. We suggest an inductive study approach to postulate a hypothesis and synthesis of supporting evidence as a trial to resolve hypoxia in patients with COVID-19 by increasing the volume of fetal hemoglobin which has a high affinity for oxygen using methods for hypothesis related research evidence synthesis.

**Conclusion:**

We recommend involving umbilical cord fetal blood transfusion or the use of hydroxyl urea as a clinical trial on COVID-19 patients and also for all other types of ARDS to determine its efficacy in correction of hypoxemia, controlling progression of the disease, and increasing survival rate.

## Background

In December 2019, a sudden public health incident (the corona virus disease [COVID-19] epidemic) occurred in Wuhan, China [1]. The disease caused by the pathogenic virus severe acute respiratory syndrome corona virus 2 (SARS-CoV-2) was named “COVID-19” by the World Health Organization (WHO) on 11 February 2020. Its original English name, “novel corona virus pneumonia,” was revised by the National Health Commission of China on 22 February 2020 to “COVID-19,” which is consistent with the WHO naming, although its Chinese name remains unchanged [2]. Today, it has spread to more than 188 countries and territories [3].

Mortality are as high as 46% in critically ill patients requiring intensive care unit admission and oxygen therapy [4], suggesting an urgent need to try therapeutic interventions in addition to supportive treatment.

Many hypotheses have explained hypoxemia in COVID-19 patients, such as hyperimmune reaction to viral infection and cytokine storm that leads to serious lung tissue and alveolar damage or even direct viral insult [3].

## Main text

### Pathogenesis of hypoxia in COVID-19

Three core pathologic processes lead to multi-organ failure and death in COVID-19:

1. A dysregulated immune system whose cells penetrate and destroy several organs, including the lungs, kidneys, and heart, is a hyper-inflammatory storm. SARS-CoV-2 is now generally known to induce aberrant T lymphocyte activation and macrophage activation, resulting in a “cytokine storm.”
2. The dysregulated immune system destroys the endothelium and stimulates blood clotting, allowing micro and macro blood clots to form hyper-coagulability (increased clotting). Blood supply is affected by these blood clots.
3. Extreme hypoxemia (low blood oxygen levels: lung inflammation caused by the cytokine storm along with microthrombosis in the pulmonary circulation severely impairs oxygen uptake, resulting in oxygen failure [5].

The above pathologies are not novel; however, the combined severity in COVID-19 disease is considerable. It is important to remember that it is not the infection that destroys the patient, but the overactive immune system of the patient. Providing supportive care (with ventilators which stoke the fire themselves) and waiting for the cytokine fire to burn itself out simply does not work [5].

### Hypothesis

#### Difference between adult hemoglobin and fetal hemoglobin and its necessity to the fetus

There is more than one type of hemoglobin. In adults, Hb A or adult hemoglobin is the main hemoglobin in the blood, but there is another type of hemoglobin called fetal hemoglobin. Fetal hemoglobin (also hemoglobin F, Hb F, or α2γ2) is the main oxygen carrier protein in the human fetus. Hemoglobin F is found in fetal red blood cells (f cells) and is involved in transporting oxygen from the mother’s bloodstream to fetal organs and tissues. It is produced at around 6 weeks of pregnancy [6], and the levels remain high after birth until the baby is roughly 2–4 months old [7]. Hemoglobin F has a different composition from hemoglobin A and higher affinity to oxygen, which allows it to bind to oxygen more strongly. This way, the developing fetus is able to retrieve oxygen from the mother’s bloodstream, which occurs through the placenta at intervillous space, where Hb F in fetal blood catches oxygen from Hb A in maternal blood [8].

At birth, hemoglobin F accounts for 50–95% of the infant’s hemoglobin and at around 6 months after birth, hemoglobin A becomes the predominant type. Levels of hemoglobin F gradually decrease and reach adult levels (less than 1% of total hemoglobin) usually within 1 year of life [9].

#### Why hemoglobin F has high oxygen affinity

The four hemes, which are the oxygen-binding parts of hemoglobin, are similar between hemoglobin F and other types of hemoglobin, including hemoglobin A. Thus, the key feature that allows hemoglobin F to bind more strongly to oxygen is by having γ subunits (instead of β, in Hb A for example). In fact, some naturally existing molecules in our body can bind to hemoglobin and change its binding affinity for oxygen. One of the molecules is 2,3-bisphosphoglycerate (2,3-BPG) and it enhances hemoglobin’s ability to release oxygen [10]. 2,3-BPG interacts much more with hemoglobin A than with hemoglobin F. This is because the adult β subunit has more positive charges than the fetal γ subunit, which attract the negative charges from 2,3-BPG. Due to the preference of 2,3-BPG for hemoglobin A, hemoglobin F binds to oxygen with more affinity, in average [11].

#### GBT1118 effect on hypoxia

In an interesting study, the therapeutic effect of two different doses of GBT1118 (a compound that enhances the oxygen affinity of hemoglobin) was evaluated in a murine model of acute lung injury induced by intratracheal lipopolysaccharides (LPS) instillation 24 h before exposure to 5% or 10% hypoxia in mice. GBT1118 administration to mice greatly improved the oxygen affinity of hemoglobin. Compared with mice receiving vehicle control, mice treated with GBT1118 had significantly lower mortality after LPS + 5% hypoxia (47% with vehicle vs. 22% with low-dose GBT1118, 13% with high-dose GBT1118) and had decreased severity of illness. Mice treated with GBT1118 showed a sustained significant increase in SpO2 over 4 h of hypoxia exposure. GBT1118 did not attenuate lung inflammation or even affect alveolar-capillary barrier permeability which means increasing survival and decreasing mortality by its ability to increase oxygen affinity to hemoglobin only [12].

#### Normal people with high fetal hemoglobin percentage

Some people have hereditary high amount of fetal hemoglobin and this condition is called hereditary persistence of fetal hemoglobin (HPFH), and it is found that people with hemoglobinopathies like sickle cell anemia or beta-thalassemia attributed with high amount of fetal hemoglobin become mostly asymptomatic or have mild symptoms [13, 14].

#### Rational of hypothesis

So, the rational of this hypothesis is that we need to increase fetal hemoglobin which has high affinity to oxygen in COVID-19 patients so we can overcome hypoxia and desaturation that cause more tissue damage and lead to cardiac arrest—as in cases with happy hypoxia which have happened widely in COVID-19 patients—thus, we buy time for the patient’s immunity to overcome the virus.

#### How can we increase fetal hemoglobin amount

We can accomplish that by two ways: (A) injecting fetal blood from umbilical cord of newly born infants which is rich with fetal hemoglobin directly to patient, or (B) giving the patient hydroxyurea which raises F cells and fetal hemoglobin.

(A) The volume of umbilical cord blood varies from 50 to 140 ml with a mean of 85 ml rich in fetal hemoglobin, the average percentage is 85.5% with a range from 79 to 91%. Cord blood is currently used safely with people suffering from varying stages and grades of malignancy, thalassemia major, aplastic anemia, systemic lupus erythematosus, chronic renal failure, rheumatoid arthritis, ankylosing spondylitis, and a geriatric group of patients with benign prostatic hypertrophy after following the precautions of standard blood transfusion protocol. All have tolerated the procedure without any immunological or non-immunological reactions [15].
(B) Hydroxyurea has been used for a long time in the treatment of myeloproliferative diseases, reducing vaso-occlusive crisis, acute chest syndrome and transfusion requirements by increasing fetal hemoglobin (Hb F) in patients with sickle cell disease, thus minimizing hemoglobin S polymerization and clinical complications [16].

The precise mechanism of action is not yet clear, but it appears that hydroxycarbamide increases nitric oxide levels, causing soluble guanylyl cyclase activation with a resultant rise in cyclic GMP, and the activation of gamma globin gene expression and subsequent gamma chain synthesis necessary for fetal hemoglobin (Hb F) production (which does not polymerize and deform red blood cells like the mutated HbS, responsible for sickle cell disease). Hydroxyurea also suppresses the development of bone marrow granulocytes that have a mild immunosuppressive effect, particularly at vascular sites where blood flow is obstructed by sickle cells, so it is used in cases of thrombocytosis and leukocytosis associated with a higher risk of thrombosis [17, 18].

Indeed, maximal increments in F reticulocytes are attained at 10 or 11 days after the start of hydroxyurea treatment in patients with sickle cell anemia [19, 20], indicating a rapid impact on the essential pathways responsible for deciding the phenotype of the globin gene.

F cell tests also indicate maximum changes within 14–21 days [21]. Induction of Hb F of similar magnitude in the cells of normal individuals (1.3- to 3.5-fold) and patients with sickle cell disease (2- to 5-fold) by hydroxyurea was already reported using a similar erythroid cell culture system [22].

### Suggested study protocol (deductive research)

#### Rationale of the study

Although many empirical therapeutic options have been introduced on several operational recommendations, including existing and new generation of antivirals, steroids, anticoagulants, and supportive management; however, the optimal management strategy for severe COVID-19 remains unclear and the degree of illness varies, ranging from asymptomatic to fulminant and fatal. While treatment of the virus itself is certainly desired, management of hypoxia which is one of the primary causes that leads to multiple organ injuries and death in COVID-19 patients may be the clue to improve the survival. High oxygen therapy has adverse toxic effect.

#### Objective of the study

To provide a clearer understanding of how increasing fetal hemoglobin directly affects the outcomes in patients with fulminant COVID-19.

#### Methodology of the study

##### Technical design

A) Site of study: this study will be conducted in COVID-19 isolated intensive care units.
B) Ethical approval and consent to participate: approval will be obtained from Institutional Review Board (IRB). Written informed consent will be signed by all patients or their first-degree relatives after discussing the study design including the procedure.

A written informed consent will be signed from all patients or their first degree relatives after discussing the study design including the procedure.

C) Sample size: a pilot study.
D) Study design: prospective randomized controlled clinical trial.

Deteriorating hypoxic patients with fulminant COVID-19 will be divided randomly into two groups:

- # Control group: patients will receive standard investigations and treatment protocol for COVID-19.
- # Protocol group: patients with trial of increasing the amount of fetal hemoglobin besides receiving standard protocol.

E) Inclusion criteria: admitted COVID-19 patients to the ICU with the following clinical criteria:
  Hypoxia with respiratory distress
  Hypoxia (need noninvasive or invasive ventilatory support)
  Refractory hypoxemia
  > 50% involvement of the lung parenchyma on chest imaging.
F) Exclusion criteria: no exclusion
G) Study outcomes
  The primary outcome of this study will be 30 days or discharge survival of selected patients. This will be defined as survival to hospital discharge or to 30 days if the patient remained in the hospital.
  Secondary clinical outcomes will include level of hypoxemia before and after intervention, oxygen perfusion parameters, certain medication usage (inotropes, vasoinotropes, and intravenous fluid administration), investigations (rate of CT scanning), ventilator days and length of ICU stay, ICU readmission rates, and cost-effectiveness.

## Conclusion

This hypothesis concentrates on one and the main dangerous morbidity that occurs in COVID-19 patients which is hypoxia, and we offer a solution by increasing fetal hemoglobin in the critical patients as a trial to impact the course of the disease and minimize the morbidity especially in moderate and sever cases who suffer from desaturation until suppression of the immune dysregulation and avoidance of the impending death. Accordingly, we explicitly recommend involving fetal blood in umbilical cord for transfusion and the use of hydroxyurea in a clinical trial on COVID-19 patients and also all other types of ARDS to determine its efficacy in controlling progression of the disease and increasing survival rate.

## Data Availability

Not applicable

## Abbreviations

COVID-19: Coronavirus disease 2019
ARDS: Acute respiratory distress syndrome
WHO: World Health Organization
SARS-CoV-2: Severe acute respiratory syndrome corona virus 2
2,3-BPG: 2,3-bisphosphoglycerate
GBT: Global Blood Therapeutics
HPFH: Hereditary persistence of fetal hemoglobin
LPS: Lipopolysaccharides
ICU: Intensive care unit
Hb F: Fetal hemoglobin
Hb S: Sickle hemoglobin
